# Computational Design of Mineral-Based Materials: Iron Oxide Nanoparticle-Functionalized Polymeric Films for Enhanced Public Water Purification

**DOI:** 10.3390/polym17152106

**Published:** 2025-07-31

**Authors:** Iustina Popescu, Alina Ruxandra Caramitu, Adriana Mariana Borș, Mihaela-Amalia Diminescu, Liliana Irina Stoian

**Affiliations:** 1 Geological Institute of Romania, 1 Caransebes Street, District 1, 012271 Bucharest, Romania; iustinapopescu@yahoo.com (I.P.);; 2 National Institute for Research and Development in Electrical Engineering ICPE-CA, 313 Splaiul Unirii, 030138 Bucharest, Romania; 3 National Research and Development Institute for Optoelectronics INOE 2000–IHP, 14 Cutitul de Argint Street, District 4, 040558 Bucharest, Romania; 4 Faculty of Energy Engineering, National University of Science and Technology POLITEHNICA Bucharest, 313 Splaiul Independentei, District 6, 060042 Bucharest, Romania; mihaela.diminescu@upb.ro

**Keywords:** composite materials, Materials Studio DFTB+, BIOVIA Pipeline Pilot, HDPE polymer composite, Fe_3_O_4_ nanopowder, molecular modeling, Cu^2+^ and Ni^2+^ ion removal

## Abstract

Heavy metal contamination in natural waters and soils poses a significant environmental challenge, necessitating efficient and sustainable water treatment solutions. This study presents the computational design of low-density polyethylene (LDPE) films functionalized with iron oxide (Fe_3_O_4_) nanoparticles (NPs) for enhanced water purification applications. Composite materials containing 5%, 10%, and 15% were synthesized and characterized in terms of adsorption efficiency, surface morphology, and reusability. Advanced molecular modeling using BIOVIA Pipeline was employed to investigate charge distribution, functional group behaviour, and atomic-scale interactions between polymer chains and metal ions. The computational results revealed structure–property relationships crucial for optimizing adsorption performance and understanding geochemically driven interaction mechanisms. The LDPE/Fe_3_O_4_ composites demonstrated significant removal efficiency of Cu^2+^ and Ni^2+^ ions, along with favourable mechanical properties and regeneration potential. These findings highlight the synergistic role of mineral–polymer interfaces in water remediation, presenting a scalable approach to designing multifunctional polymeric materials for environmental applications. This study contributes to the growing field of polymer-based adsorbents, reinforcing their value in sustainable water treatment technologies and environmental protection efforts.

## 1. Introduction

The provision of safe, potable water represents one of the most significant challenges for contemporary society, exacerbated by accelerating industrialization and demographic growth [1,2,3]. Unregulated or inadequately treated effluents from industrial and municipal sources have led to the accumulation of toxic and hazardous substances in surface and groundwater bodies [4,5], posing significant risks to both the environment and human beings. Consequently, the development of robust treatment schemes capable of removing or degrading these pollutants is of paramount importance [6,7]. Traditional physicochemical and biological remediation methods have limited efficacy, high operational costs, and the potential to form secondary pollutants [8,9,10]. Thus, as nanomaterials developed for environmental applications, magnetic NPs offer additional advantages, such as their ability to be produced on a large scale, low toxicity, and eco-compatibility [11,12,13,14,15,16,17]. The most common magnetic NPs are composed of metals, as well as their oxides, such as nickel, iron, and cobalt [18,19,20].

Among the various mineral materials studied, such as clays, zeolites, aluminosilicates, sulphates, and iron hydroxides, zero-valent iron (ZVI) NPs have demonstrated exceptional performance [21]. Through electron transfer processes with adsorbed contaminants, ZVI facilitates advanced reductive degradation of organic compounds and transforms metal ions into less soluble oxidation states, thus promoting contaminant sequestration.

Through electron transfer processes with adsorbed contaminants, these NPs facilitate the advanced reductive degradation of organic compounds and transform metal ions into less soluble oxidation states, thus promoting contaminant sequestration. Naturally dissolved iron in water is oxidized, either by dissolved oxygen or by residual disinfectants such as chlorine, to form ferric hydroxide precipitates. These precipitates induce turbidity and promote the accumulation of iron oxide crusts in distribution networks. Such a crust is particularly exacerbated during periods of stagnation, for example, following maintenance-induced flow interruptions or hydraulic redirection. At the same time, the interaction of water laden with pollutants can accelerate the degradation of pipe materials (ductile iron (DF), high-density polyethylene (HDPE), or glass-fibre-reinforced polyester with sand incrustations (GFRP)) and can compromise hydraulic integrity. For pipes made of polymeric and composite materials, additional protective measures are required to safeguard against mechanical and chemical damage. Damage to DF distribution networks often occurs due to the solubilization of iron salts in acidic environments and mineral deposits, as well as microbiological corrosion, which accelerates bacterial colonisation [21,22].

Progressive deterioration driven by dissolved iron species—ranging from ferrous (Fe^2+^) to ferric (Fe^3+^) forms—has a negative impact on water quality (taste, odour, colour, and turbidity), while promoting particulate and biofilm formation that impacts treatment efficacy [23].

As is known, iron oxides exist in different crystalline phases, such as magnetite (Fe_3_O_4_), maghemite (γ-Fe_3_O_4_), and hematite (α-Fe_3_O_4_), magnetite being the most widespread type [24] with the highest magnetism of all iron oxides. Maghemite, on the other hand, is a mixture of the composition and structure of magnetite and hematite, reflecting structural similarities of both forms of iron oxides. γ-Fe_3_O_4_ is a reddish-brown material that forms on soil due to the corrosion of magnetite, and α-Fe_3_O_4_ is the oldest known form of iron oxide [25].

Some recent studies have used magnetic NPs to help remediate oil-contaminated soils [26,27]. The use of Fe_3_O_4_ NPs could reduce reliance on conventional methods, thereby reducing treatment costs and environmental risks [28].

In this work, a computational simulation was used to predict and optimize the interaction, at the molecular level, between Fe_3_O_4_ NPs and target pollutants, providing insights into the synthesis of polymer composite films with water purification applications and improved catalytic and adsorption properties. Thus, by comparative analysis of the coupling of geologically analysed polymeric material selection with a computer simulation, we aim to establish a scalable and efficient platform for the mitigation of emerging contaminants in public water supplies [29]. This analysis allows us to directly correlate the predictions of the computer simulation (e.g., binding energies, adsorption sites, and electronic structure changes) with the experimental observations obtained from SEM, EDX, and BET analyses and aims to confirm the effectiveness of the material in capturing and degrading contaminants. Thus, this work aims to develop a method for purifying tap water, associated with algorithms that can provide a complete picture of its contamination, in terrestrial areas, presenting certain advantages over the other methods studied to date [30,31,32,33].

This approach is helpful in identifying areas requiring intervention, as well as in establishing criteria for preventing exposure to contaminated water. The deposition of ferric oxide precipitates and other chemical contaminants, including synthetic organic pollutants, on distribution network pipes adsorbs the residual chlorine from the water, thus reducing the chlorination effect. Therefore, it is necessary to monitor water parameters to maintain them within the permissible limits. By comparative analysis of the efficiency of wastewater decontamination using LDPE/Fe_3_O_4_-type polymeric films through computer modeling (DFT, DFTB+) and experimental validation (SEM, EDX, BET), it was demonstrated that these materials maintain their structural integrity, offer high magnetic reactivity, and exhibit improved adsorption of heavy metal ions [34,35,36,37].

Computer simulations highlight the electronic and magnetic properties of Fe_3_O_4_ NPs embedded in the polymer matrix, which lead to an increase in the affinity of the composite for heavy metals and organic pollutants. Experimental tests validate these predictions by demonstrating the effectiveness of the chosen solution, establishing a clear link between the computer simulation and the experimental results obtained. Water abstracted from the aquifer is often contaminated simultaneously with several pollutants, which can interact, resulting in profound effects on human health; therefore, the choice of material for replacing pipes and its adaptation to the network connection are issues that are intensively studied. The novelty of this work consists in the selection (after a careful and detailed analysis of these materials) and treatment of the polymeric composite material for an improvement of its anti-corrosive characteristics.

The anti-corrosion coating, made of a blend of low-density polyethylene (LDPE) and Fe_3_O_4_ NP filler (NPFE), exhibits strong adhesion, low shrinkage, and good chemical stability in water. The role of the filler (Fe_3_O_4_ NPs) is to improve the adhesion of the polymer film and its durability and to prevent microcracking.

Overall, the synergy between computer modeling and experimental validation allows for a more rational and efficient design of polymer composites tailored for enhanced water purification, providing an understanding of the mechanisms that occur and how these materials interact with contaminants and improve water quality.

## 2. Materials and Methods

### 2.1. Materials

The following raw materials were utilized in this study:

- Commercial low-density polyethylene (LDPE) powder—BRALEN NA 7–25—from BASTPLAST SRL, Bucharest, having the characteristics presented in Table 1.
- Compatibilizer for polymer mixtures: Admer NF 468E polyethylene grafted with maleic anhydride (LDPE-g-MA) from Mitsui Chemicals Europe GmbH, Germany [38].

**Table 1:** LDPE characteristics.

Properties	UM	Test Method	Typical Value
Density (23 °C) *	kg/m^3^	[39]	915
MFR (190 °C/2.16 kgf)	g/10 min	[40]	8
Tensile strength (MD/TD) **	MPa	[41,42]	17/16
Tensile strain at break (MD/TD) **	%	[41,42]	350/550
Vicat softening temperature *	°C	[43]	84
Shore D hardness *	-	[44]	42

* Average mechanical property values of several measurements carried out on standard injection-molded test specimens prepared in accordance with [45]. ** Properties on film in MD/TD—thicknesses of 0.07 mm (MFR = 0.35 g/10 min) and 0.04 mm (MFR more than 0.35 g/10 min).

- Fe_3_O_4_ magnetic nanopowder purchased from Nanografi [46,47] with 99.55% purity. The characteristics of Fe_3_O_4_ nanopowder are presented in Table 2.

### 2.2. Methods and Equipment

#### 2.2.1. Determination of Adsorption Characteristics

Adsorption characteristics were determined using the MaterialsScript API program for materials science in complement to laboratory experiments; thus, changes in the free energy of these structural geometries were identified. The efficiency of the photosensitive layer (EPL) and the electric potential in the circuit (eVoc) of the functional groups of metal ions existing in public water were also evaluated. The method offers the possibility of analyzing the type and nature of the electronic transition in intramolecular charge transfer, as well as the amount of charge transferred, at the structural level. The accuracy of the properties in the chemical reaction of the molecules is given by the particle size—density functional theory, scaling, and the nature of the repetitive charges (location of the transition states)—and accessing the data that can provide the system energy of the configuration is carried out with the Materials Studio DMol3 module. From the analysis of these physicochemical simulation data of adsorption–desorption and diffusion processes, we determined the role of functional groups at the microscale for their surface displacement and the gradient tensors of the field-bounded action field in a multilinear manner that prove the behavior of polymeric structures as a function of temperature, yield limit, and critical deformation.

#### 2.2.2. Obtaining Polymer Composite Materials

The LDPE polymer was first mixed with the LDPE-g-MA compatibilizer, and then Fe_3_O_4_ powder was added. The resulting blend was subjected to ultrasonication for 30 min to promote uniform dispersion and prevent clumping of the magnetic NPs. The mixture, hence formed, was injected from the melt. The injection was performed on Dr. Boy injection moulding machine (Neustadt-Fernthal from Germany) with the following characteristics: a maximum clamping force of 350 kN and a maximum working temperature of 300 °C, with the diameter of the ladle being 28 mm and the L/D ratio being 18.6. Obtaining these composites was performed similarly to that in [48]. The processing temperature range across the five heating zones of the machine is 190–220 °C for LDPE polymer matrix composites. Eight concentrations of LDPE/Fe_3_O_4_ nanopowder composites were obtained using Fe_3_O_4_ nanopowder mass percentages of 5, 10, and 15%. Several concentrations were obtained to identify the effect of Fe_3_O_4_ nanofiller. Specimens were obtained for structural, mechanical, dielectric, and thermal characterization. The concentrations and codifications of the obtained polymer composite materials expressed by the ratio of LDPE/LDPE-g-MA/Fe_3_O_4_ nanopowder are presented in Table 3.

From the composite material with the best properties (M5 coded sample), a film was made with a SPECAC High Temperature Film Maker (220 V, 50 Hz) with a temperature range of 20–400 °C. The machine is capable of producing films of in-range thickness (15–500 μm) from 10 mm diameter samples.

#### 2.2.3. Photodynamic Molecular Simulations Carried out for the Determination of the Adsorption Characteristics

The metals considered are Fe(II), Ni(II), or Cu(II). Thus, the characteristics, adsorption behavior of ions, and active functional groups of the metals mentioned on the surface and at the interface of the liquid medium with the column surface were determined by the Modeling & Simulation for Next-Generation Materials module of BIOVIA Materials Studio software (v. 5.5.2/2017). The calculated adsorption energy values are predominantly negative, indicating that adsorption is likely to be spontaneous. Also, the values determined with the BIOVIA Pipeline Pilot specification show the dispersion and interaction mode of the functional group geometries of the polymer film, as well as that of the excitation energies of ions in the energy-maximized functional positions.

The determination of large, complete data sets, generated for a specific period and a specific monitored region, provides the necessary leverage and means to apply an efficient testing and analysis method with accuracy in terms of the presence and quantity of pollutants of interest. At the same time, the quantitative determination of pollutant mixtures facilitates informed decision making in various situations, depending on their respective regulatory limits. Based on data visualization algorithms that facilitate decision making by authorities, the need for continuous monitoring measures has been established. This may help improve prevention and maintenance measures for both groundwater quality and water quality in public supply networks. At the same time, these associated methods (algorithms and additional data) can provide a comprehensive view of water contamination in the analyzed terrestrial areas. This approach can be helpful not only in identifying areas in need of intervention but also in establishing criteria to prevent public exposure to potentially contaminated water.

#### 2.2.4. Magnetic Testing

Static investigation of the magnetite oxide nanopowders was carried out using a hysteresis graph, and the data were collected in a closed magnetic circuit. Magnetic characterization was carried out on a Lake Shore VSM-7304 vibrating sample magnetometer (VSM) VSM-7304; magnetic tests consisted in performing comparative hysteresis cycling for M1–M8 (materials with varying filler concentrations of 5%, 10%, and 15%, respectively). Each sample was prepared as a hollow cylinder with an outer diameter of 10 ± 0.1 mm, an inner diameter of 8 ± 0.1 mm, and a thickness of 2 ± 0.1 mm to measure the inductance at the ends of the wire. When the cylindrical sample is inserted into the test assembly, an ideal, single-turn inductor is formed without flux loss. Inductance measurements were performed in the frequency range of 10–33 MHz.

#### 2.2.5. Structural Analysis

The microparticles structure and morphology were characterized by SEM, using Quanta Inspect F50, FEI Company, Eindhoven, The Netherlands, which was equipped with a field emission electron gun (FEG) with a resolution of 1.2 nm and an energy-dispersive X-ray spectrometer (EDS) with a resolution of MnK of 133 eV. The microparticles structure and morphology were characterized by SEM, using Quanta Inspect F50, FEI Company, Eindhoven, The Netherlands, which was equipped with a field emission electron gun (FEG) with a resolution of 1.2 nm and an energy-dispersive X-ray spectrometer (EDS) with a resolution of MnK of 133 eV.

## 3. Results

### 3.1. Characteristics of the Deferrization Treatment

The samples collected from the water distribution network in the analyzed area were treated at the laboratory level, and the experimental results obtained indicate a fluctuation in corrosivity and provide useful information for monitoring: the corrosion rate in the distribution network and water quality. Turbidity is a crucial index for assessing the quality of drinking water in terms of the degree of obstruction to the passage of light through a solution. This phenomenon is closely related to the content of impurities, such as sediment, clay, plankton, and microorganisms [49]. The characteristics of deionized water and the recorded turbidity data are presented in Table 4.

Therefore, the water is delivered with the required drinking water characteristics, including turbidity below the maximum permissible limit of 5 NTU [50]. The life cycles of functional groups and excited ions produce significant stress on the polymer structure, which can be highlighted with the BIOVIA Materials Studio DFTB+ simulation program, based on the functional density and electronic properties of these groups.

Using the method configured by the Materials Studio DFTB+ model, defects and complex interactions of atomic orbitals between the organic and inorganic surfaces of the composite were studied. The recorded parameter values in the DFTB+ database were compared with the Slater–Koster parameter libraries, which contain specific values of the interactions between the reactive elements in the analyzed polymer composite material. If elements appear that are not identified, the Materials Studio DFTB+ module provides specific comparative data or support for the development of new sets of values, allowing the extension of experimental data to new systems that detailed predictions can analyze. In addition to the usual repulsive and short-range repulsive electronuclear terms, the Kohn–Sham approximate final energy additionally includes a Coulomb interaction between charge fluctuations. Electrostatic forces are considered between two-point charges at varying distances and also include self-interaction of atoms if the charges are located on the same atom.

### 3.2. Determination of Optimal Molecular Geometries

Based on the molecular dynamics of different optimized geometries, one can determine the density of the polymer band, the energy of the orbitals in the layers, and some mechanical properties of the durable structure.

The oxidation potential of excited spinel molecules for the calculation of photosensitization adsorption parameters is given by the following relation:

P_ox_ = M_ex_ − λ_max_ + E_photo_ (1)

where:

P_ox_ = oxidation potential.

λ_max_ = the wavelength of maximum adsorption of the lowest-energy electronic transition in the UV/vis adsorption spectrum of the excited molecule (Mex), cumulated with the photosensitizing energy (E_photo_).

eV_photo_ = E_photo_ − Γ (2)

where:

Γ = band dipole moment correction factor.

EPL = 1 − log F × η(3)

where:

EPL = photosensitive coating efficiency.

eV_photo_ = open-circuit photo voltage correlated with the level of sensitization, as follows:

F = oscillating power as the corresponding UV/vis adsorption band value.

η = overall efficiency, η as a function of photosensitive cell energy.

The optimal molecular geometries for the polymer film structures were calculated using approximate estimates of solvation energies, calculated for metal ions as the difference between the total energies in solvents used for recovery from the residual effluent (tetrahydrofuran (THF) and ethanol (EtOH)) and the solution, and are reported in Table 5. Simulations have complemented experimental data, providing new insights into polymer–turbulence interactions, in particular the suppression of turbulent vortices by elastic stresses [51,52]. Tests performed in flow systems (pipes and channels) have clarified the interaction between turbulence modulation and fluid elasticity, correlating pressure drop, velocity profiles, and elastic shear stress with the efficiency of flow resistance reduction [53,54].

The values on the energy shift of solvents (essential in modifying the photophysical properties) lead to increased electronic excitation in adsorption reactions in the photosensitization processes of functional groups and lattice ions, which is in agreement with UV/Vis spectra and the adsorption–desorption isotherms of metal ions and some functional groups. The eV_photo_ values indicate that the more significant the adsorption–desorption phenomenon, the more efficient the sensitization, so the solvent has a smaller effect on the charge transfer. The calculated average intramolecular charge value of 0.68 indicates that there is an equilibrium at the transitions and that this could be associated with possible defects in the structure when there is a tendency for the energy to increase on specific facets of the structure. Thus, spontaneous deformation energies appear at the surface of structures, including functional groups and excited ions, which means that the solvent has an increased effect on the functional structures. Possible adsorption configurations may occur at the interaction between the two surfaces of the organic–organic system as the temperature slowly rises. The additional local energy phenomenon shows an increased adsorption of carboxylate functional groups or oxido-metallic groups, marked by their adsorption energies at the surface of the configurations. The adsorption and deformation energies indicate that there are configurations of adsorbed functional groups that are influenced by the excitation energies and their direction of adhesion (horizontal or vertical), and they also suggest that their adsorption can be spontaneous. Still, the direction of adsorption–desorption is a function of the nature of the functional group, the pore-filling gradient, and the temperature.

### 3.3. Magnetic Tests

**Figure 1:**
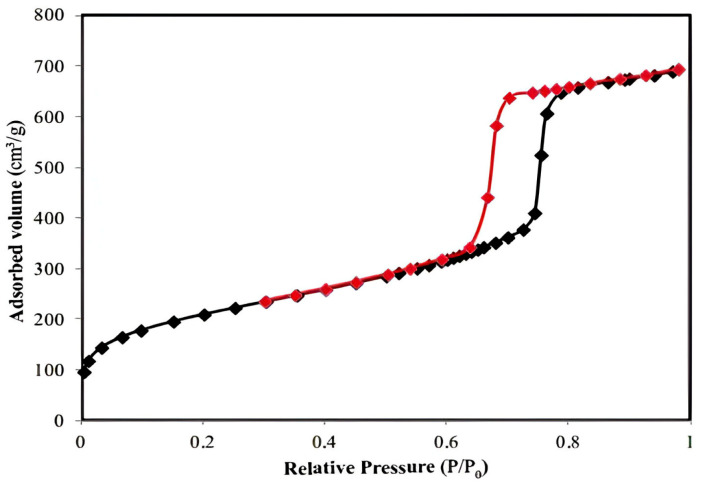
Adsorption–desorption isotherms of Fe ions and functional groups on the analyzed polymer film; red curve denotes the desorption, black curve denotes the adsorption.

The hysteresis loop is characteristic of ordered mesoporous solids with a stable pore geometry, and the steep configuration suggests that the Fe_3_O_4_ polymeric film structure has a narrow pore size distribution—the Barrett–Joyner–Halenda (BJH) model—which, according to the literature [55], increases their relative filling pressure (Figure 1).

### 3.4. Structural Analyses

**Figure 2:**
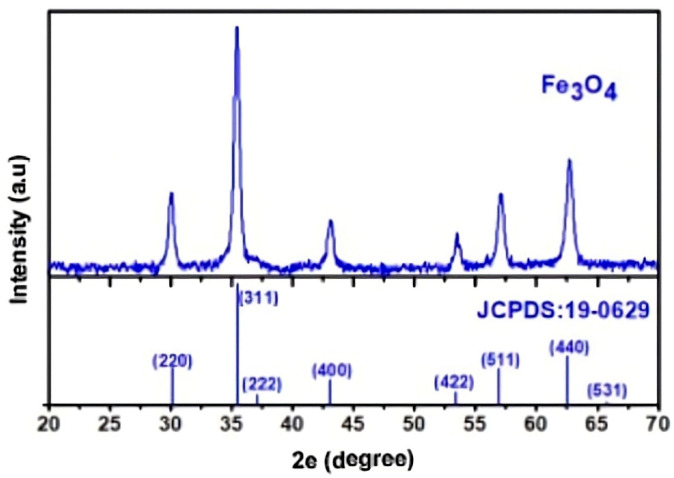
Structural image of the magnetite (Fe_3_O_4_) analyzed and a diffractogram for standard magnetite according to JCPDS: 19-0629.

SEM was used to characterize the morphology of oxide nanopowders (Fe_3_O_4_) after adsorption of Cu^2+^ and Ni^2+^ ions from contaminated aqueous solutions (Figure 2). The composition of these metal ion-loaded powders is highlighted by the EDX spectrum (energy-dispersive X-ray spectroscopy). It demonstrates that the process of metal ion incorporation and adsorption into the oxide powder structure results in the formation of complex structures.

**Figure 3:**
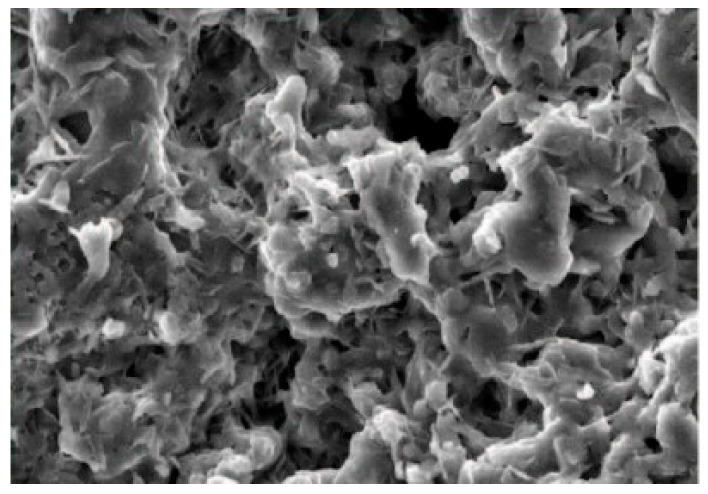
SEM micrograph of Fe_3_O_4_ NPs samples loaded with metal ions.

**Figure 4:**
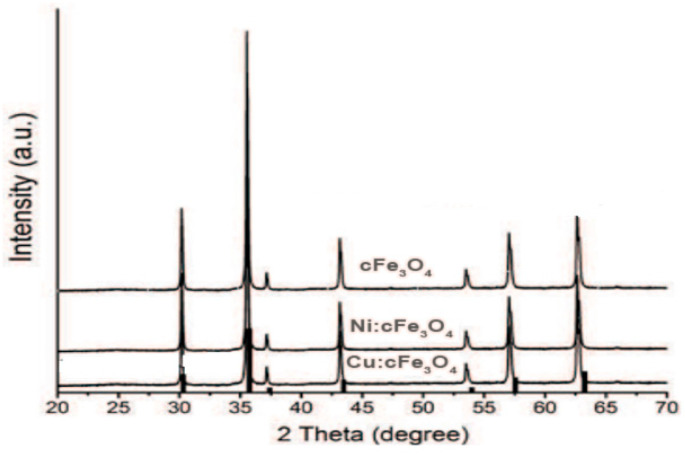
X-ray diffraction spectra of Fe_3_O_4_ oxide nanopowders loaded with Ni^2+^ and Cu^2+^ ions.

The authors also had concerns in performing SEM analyses of polymer composite materials with metal fillers [56,57,58] (Figure 3), as well as in the effect of the extremely low-frequency electric field on living matter in wastewater [59,60]. The loading with metal ions of the Fe_3_O_4_ oxide nanopowder is shown in Figure 4.

Table 6 shows date regarding the magnetic measurements and EDX analyses.

**Figure 5:**
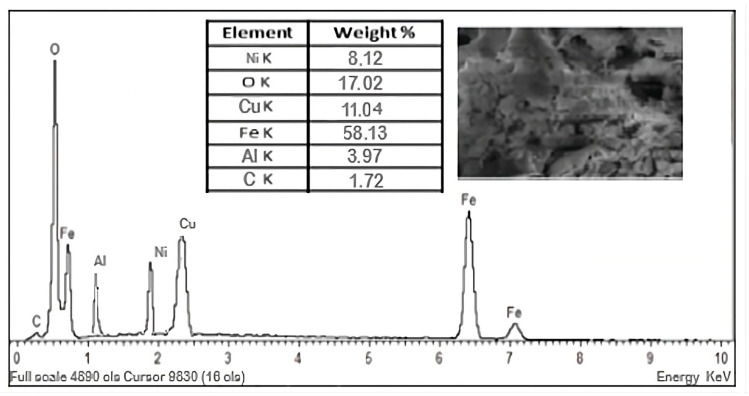
EDX spectrum of magnetite (Fe_3_O_4_) NP samples loaded with metal ions.

EDX analysis of these nanopowders indicates the presence of oxygen, iron, nickel, copper, and aluminum (Figure 5). Additionally, the presence of a minor peak for carbon (C) was observed in the EDX spectrum.

The interaction of nickel and copper ions with the iron oxide structure led to a shift in the spinel structure of Fe_3_O_4_. The average particle size of Cu: Fe_3_O_4_ and Ni: Fe_3_O_4_ is 78 ± 0.2 nm and 11 ± 0.1 nm, respectively. The spherical morphology of the Fe_3_O_4_ oxide structure presents elongated, irregular shapes, which shows that the adsorption of heavy metal ions influences the oxide shape of the powder, and the pore size is slightly different according to the BET isotherm (relative pressure P/P: 0.001–1 mmHg). Cu and Ni ions immobilized by the Fe_3_O_4_ nanopowder were detected by EDX analysis. Adsorption kinetics were performed by varying the concentrations of Ni^2+^ and Cu^2+^ in the solution to be analyzed to evaluate the decontamination efficiency of the Fe_3_O_4_ nanopowder sample.

**Figure 6:**
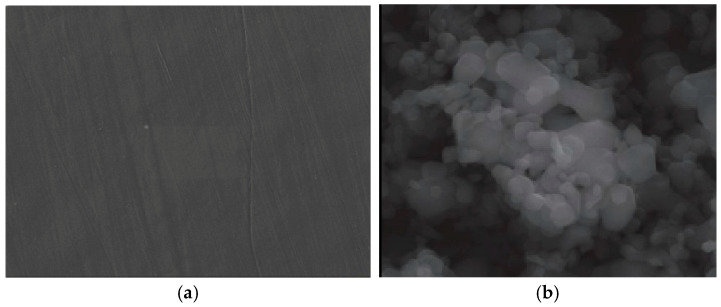
SEM micrograph of (**a**) LDPE and (**b**) oxide sample loaded with copper ions (Cu: Fe_3_O_4_).

**Figure 7:**
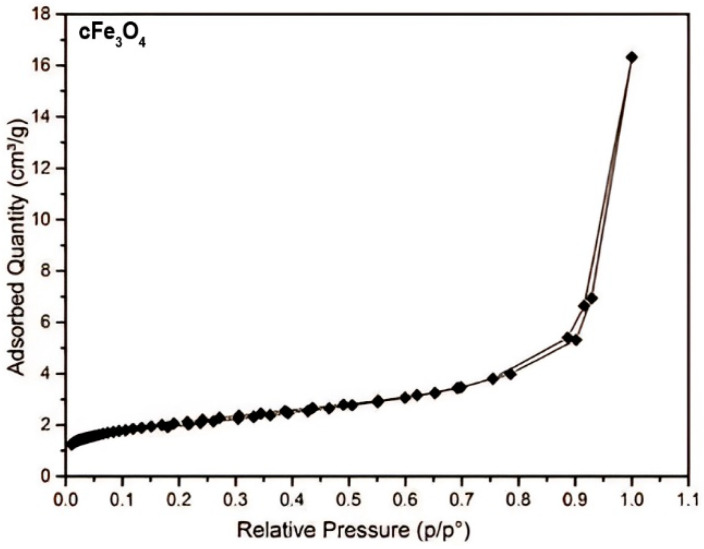
Adsorption/desorption isotherm of Fe_3_O_4_ nanopowder.

In Figure 6, the micrographs for the raw materials used were presented, namely the LDPE polymer (Figure 6a) and Fe_3_O_4_ (Figure 6b). The Figure 7 shows isotherme of adsorption-desorption of Fe_3_O_4_ nanopowder, and the Table 7 shows characteristics date about LDPE-g-MA compatibilizer polymer. 

**Figure 8:**
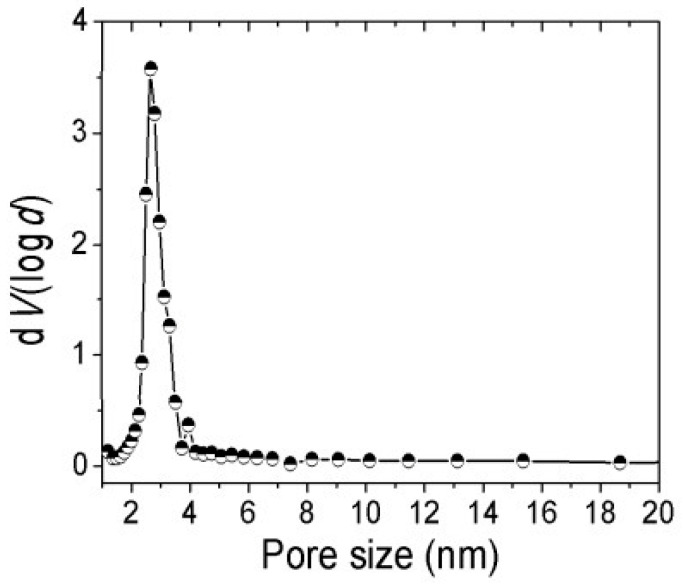
Pore size distribution for Fe_3_O_4_ NP/LDPE-g-MA.

Distribution of the pore size of the Fe_3_O_4_ oxide nanopowder and compatibilizer polymer (LDPE-g-MA) are graphically represented in the Figure 8.

The experiments conducted led to the determination of kinetic parameters for Ni^2+^ and Cu^2+^ ions, with densities of 6.23 g/cm^3^ and 9.64 g/cm^3^, respectively. This resulted in a filling area per cation of 0.0533 nm^2^ and 0.722 nm^2^, respectively.

At the laboratory level, tests were conducted on the polymeric composite material film, and composite M5 was chosen, as it exhibited the best properties in terms of water turbidity (treated in five columns of different sizes). The determined average specific surface area of the polymer composite film was 0.2–0.6 m^2^/g for the removal of ions from tap water in columns with maximum turbidity between 11.64 and 5.33 NTU (above the maximum accepted limit) and 0.55–1.05 m^2^/g for columns with turbidity values between 5.33 and 2.86 NTU (values within the maximum accepted limit according to legislation).

Depending on the position of the ions, they form nonmagnetic spinels or ferrites with ferromagnetic spinels. 

The specific particle surface area, optical properties, and high magnetic susceptibility enable these structures to have multiple industrial applications, as well as applications in water purification and soil decontamination. The use of a polymer or surfactant coating prevents the oxidation and agglomeration of these particles. Thus, the properties of iron oxide, the functionalization of the magnetite (Fe_3_O_4_) structure, are chemically stable and can be used in various fields due to its biocompatibility.

The analysis of the obtained results corroborated with the data of the specialized studies [61,62,63,64,65,66]. It highlights the fact that iron oxide particles can be used for the decontamination of contaminated waters, the removal of toxic elements from network waters, and/or the remediation of contaminated soils. The results obtained demonstrate that the use of polymeric composite material films, made from M5 material, provides an efficient and stable chemical structure for removing heavy metals from network waters and treating groundwater.

## 4. Conclusions

The behavior of iron oxides, which exhibit characteristics similar to those of polymeric materials, as well as their synergistic combination for the development of new water decontamination techniques and environmental conservation, was analyzed in comparison.

This study demonstrates the strong potential of functionalized spinel ferrites, particularly doped with transition metals (Cu^2+^, Ni^2+^), for use in wastewater treatment.

Through computational modeling (DFT, DFTB+) and experimental validation (SEM, EDX, BET), it was shown that these materials maintain structural integrity, offer high magnetic responsiveness, and exhibit enhanced adsorption of heavy metal ions.

Key findings reveal that the adsorption mechanism in the polymer matrix varies depending on the adsorbed cation, as shown by the results of laboratory analytical methods and determinations obtained with the BIOVIA Pipeline Pilot specification.

The magnetite coated with LDPE polymer film proved effective in reducing water turbidity and capturing heavy metals.

Overall, the integration of advanced material design with geochemical insights supports the application of spinel ferrites as sustainable, high-performance agents in water purification and environmental protection.

Iron oxide MNPs are the most preferred nanomaterials in medical sciences, due to their features of minimal toxicity and excellent physicochemical properties such as super paramagnetism, stability in aqueous solutions, and biocompatibility [67,68,69,70]. In contrast, while zero-valent iron NPs (nZVI) are highly reactive and effective in some water treatment contexts, studies have shown that their application, especially at high concentrations, may raise concerns regarding environmental safety and potential toxicity [67].

Therefore, our choice to use Fe_3_O_4_ NPs was motivated by the goal of developing a more environmentally friendly and stable composite material that retains high reactivity and adsorption efficiency while reducing the potential risks associated with nZVI use.

## Figures and Tables

**Table 2:** Characteristics of Fe_3_O_4_ magnetic nanopowder.

Properties	UM	Typical Value
Size	nm	14–29
Density (23 °C)	g/cm^3^	5.18
Structure		Inverse-polyhedral-cubic
Dimensional distribution		Relatively uniform
Saturation magnetization	emu/g	27–48
Surface		Hydrophobic, biocompatible
Colloidal stability		High
Solubility		Dissolves much faster than other iron oxides
Hardness		5.5 (being similar to glass at ambient temperature)
Melting temperature	°C	1590
Boiling temperature	°C	2623
Fusion energy	kJ/mol	138.16 at a high temperature of 2623 °C
Decomposition energy	kJ/mol	138.16 at a high temperature of 2623 °C
Magnetic properties		excellent

**Table 3:** Concentrations and codifications of the composites under study.

Samples	wt.% LDPE	wt.% LDPE-g-MA	wt.% Nano Fe_3_O_4_
M1	90	5	5
M2	85	5	10
M3	80	5	15
M4	90	5	5
M5	85	5	10
M6	80	5	15
M7	85	5	10
M8	80	5	15

**Table 4:** Characteristics of the applied treatment: deferrization of the analyzed samples.

Characteristics		Treatment Method—Deferrization at the Laboratory Level					
Maximum volume flow	m^3^/h	8	16	24	32	40	
Polymer film diameter	mm	25	50	80	100	120	
Column/tube length	mm	600	1000	1200	1800	2200	
Column loading	L	14	28	32	90	180	
Initial turbidity	mg/L	20.71					
After treatment turbidity	mg/L	11.64	8.67	5.33	4.20	2.86	(T = 23 °C)

**Table 5:** Calculation of the optimal molecular geometries of the analyzed ions as a function of solvation energies.

Me^2+^	Solvent	Γ	eV_photo_	∆E (Solvent) (kcal/mol)	Adsorption Energy (kcal/mol)	Deformation Energy
Fe^2+^	EtOH	4.73	−6.21	−17.55	28.36	2.78
	THF	4.38	−6.16	−15.41		
Ni^2+^	EtOH	3.68	−5.88	−27.51	18.22	1.49
	THF	3.49	−5.92	−25.16		
Cu^2+^	EtOH	4.83	−6.14	−26.33	9.64	1.53
	THF	4.62	−6.09	−22.40		

**Table 6:** Saturation magnetization (Ms), magnetic susceptibility (χ_g_), and Fe_3_O_4_ content calculated from magnetic measurements or EDX analysis.

Sample	Ms (emu/g)	Χ_g_10^6^	Fe_3_O_4_ Content of Ms (wt. %)	Fe_3_O_4_ Content from EDX (wt.%)
Fe_3_O_4_ NP	53.21	11.00	-	-
Fe_3_O_4_ NP/LDPE-g-MA	4.19	1.38	7.7	6.8

**Table 7:** Characteristics of pore and the elemental cell.

Sample	a_0_ (nm)	S_BET_ (m^2^/g)	D_por_ (cm^3^/g)	V_por_ (nm)
Fe_3_O_4_ NP	0.751	63.9	-	**-**
Fe_3_O_4_ NP/LDPE-g-MA	4.86	488	0.41	2.57

a_0(LDPE-g-MA)_ = 0.51 nm; S_BET_ = specific surface area; D_pore_ = average pore diameter; V_pore_ = total pore volume.

## Data Availability

Data are contained within the article. Further inquiries can be directed to the corresponding authors.
